# *Caenorhabditis elegans* MES-3 is a highly divergent ortholog of the canonical PRC2 component SUZ12

**DOI:** 10.1016/j.isci.2022.104633

**Published:** 2022-06-17

**Authors:** Berend Snel, Sander van den Heuvel, Michael F. Seidl

**Affiliations:** 1 Theoretical Biology & Bioinformatics, Department of Biology, Faculty of Science, University, Utrecht, Padualaan 8, 3584 CH Utrecht, the Netherlands; 2 Developmental Biology, Department of Biology, Faculty of Sciences, Utrecht University, Padualaan 8, 3584 CH Utrecht, the Netherlands

**Keywords:** Biochemistry, protein, Bioinformatics, 3days reconstruction of protein, sequence homology

## Abstract

Polycomb Repressive Complex 2 (PRC2) catalyzes the mono-, di-, and trimethylation of histone protein H3 on lysine 27 (H3K27), which is strongly associated with transcriptionally silent chromatin. The functional core of PRC2 is highly conserved in animals and consists of four subunits. One of these, SUZ12, has not been identified in the genetic model *Caenorhabditis elegans*, whereas *C. elegans* PRC2 contains the clade-specific MES-3 protein. Through unbiased sensitive sequence similarity searches complemented by high-quality structure predictions of monomers and multimers, we here demonstrate that MES-3 is a highly divergent ortholog of SUZ12. MES-3 shares protein folds and conserved residues of key domains with SUZ12 and is predicted to interact with core PRC2 members similar to SUZ12 in human PRC2. Thus, in agreement with previous genetic and biochemical studies, we provide evidence that *C. elegans* contains a diverged yet evolutionary conserved core PRC2, like other animals.

## Highlights

• The canonical PRC2 component SUZ12 was seemingly absent in *Caenorhabditis elegans*
• Sensitive similarity searches and structure prediction unearth hidden homology
• *C. elegans* MES-3 is a highly divergent ortholog of SUZ12
• MES-3 interacts with core *C. elegans* PRC2 subunits, resembling SUZ12 in human PRC2

## Graphical abstract

**Figure:**
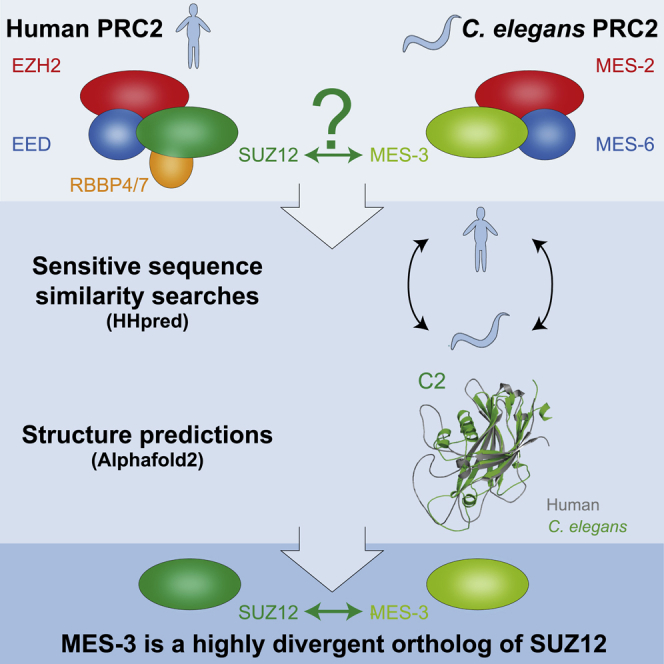

## Introduction

Posttranslational modifications of histone proteins contribute to the organization of genomic DNA and establishment of transcriptionally active versus silent chromatin (Bannister and Kouzarides, 2011). Polycomb group proteins form an important class of transcriptional repressors that function through modification of histone tails (Grossniklaus and Paro, 2014; Margueron and Reinberg, 2011). These proteins assemble into two distinct multi-subunit complexes, Polycomb Repressive Complex 1 and 2 (PRC1 and PRC2) (Bannister and Kouzarides, 2011; Bieluszewski et al., 2021; Grossniklaus and Paro, 2014; Margueron and Reinberg, 2011; Simon and Kingston, 2009). PRC2 catalyzes the mono-, di-, and trimethylation of histone protein H3 on lysine 27 (H3K27), which is strongly associated with transcriptionally silent chromatin and plays an important role in the maintenance of cell identity and developmental regulation of gene expression.

**Figure 1:**
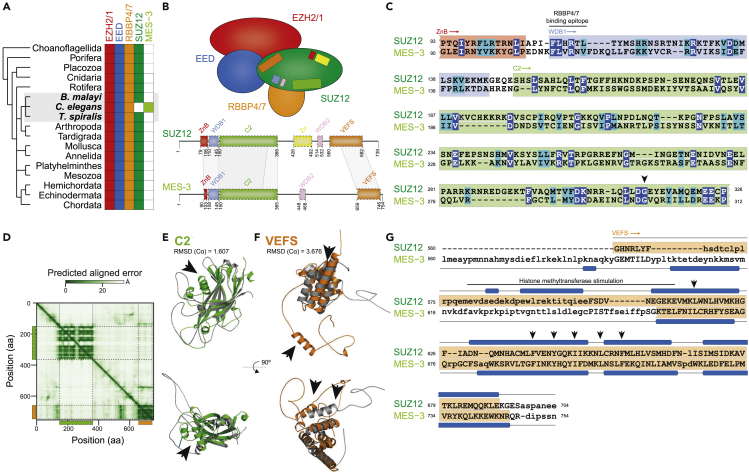
MES-3 is a highly divergent ortholog of the canonical Polycomb Repressive Complex two component SUZ12 (A). The Polycomb Repressive Complex 2 (PRC2) core components EZH2/1, EED, RBBP4/7, and SUZ12 are conserved in a broad range of metazoans; the presence of orthologs is indicated by filled boxes. Notably, based on sequence similarity searches, an ortholog of SUZ12 is absent in the nematode model species *Caenorhabditis elegans*, but present in other, closely related nematodes (*Brugia malayi* and *Trichinella spiralis*). *C. elegans* encodes the PRC2 core component MES-3 that lacks obvious motifs or sequence similarity to SUZ12 (Ahringer and Gasser, 2018; Bender et al., 2004; Ketel et al., 2005; Paulsen et al., 1995; Xu et al., 2001). (B). Schematic representation of the composition of the core PRC2. The zinc finger binding (ZnB; red), WD-domain binding 1 (WDB1; blue), C2 domain (green), zinc finger (Zn; yellow), WD-domain binding 2 (WDB2; pink), and VEFS (orange) motifs or domains involved in SUZ12 protein-interactions are shown in the schematic as well as along the protein sequence (Chammas et al., 2020; Chen et al., 2018; Kasinath et al., 2018). Schematic representation of the protein sequence of MES-3 is shown, and regions of uncovered sequence (c) and structural (e, f) similarity are highlighted. (C). Protein sequence alignment between the N-terminal region of SUZ12 and MES-3, as identified by sensitive profile-vs-profile sequence similarity searches, covers part of the zinc finger binding (ZnB; red), WD-domain binding 1 (WDB1; blue), and C2 domain (green). The conserved RBBP4/7 binding epitope as well as Gly299 are highlighted (Birve et al., 2001; Rai et al., 2013; Schmitges et al., 2011). Identical amino acids are shown in blue and biochemically similar amino acids are shown in turquoise. (D–F). The predicted aligned error (in Å; based on model 2 ptm) of the MES-3 structure is shown as a heatmap and reveals two separated globular regions in the N- and C-terminus, the former overlaps with the profile-vs-profile match (c) and corresponds to the C2 domain of SUZ12 (e; Figure S1I; RMSD = 1.607), while the latter overlaps with the region that structurally resembles the VEFS domain (f; Figure S1J; RMSD = 3.676). The black arrows (e, f) highlight regions that differ considerably between SUZ12 and MES-3 (Figures S1I and S1J), and the structure predictions of SUZ12 and MES-3 (e, f) are shown in gray as well as green (C2) and orange (VEFS), respectively. (G). Sequence-independent structure alignment of the VEFS regions of SUZ12 and MES-3 reveals significantly structural similarity (Dali *Z* score = 8.3; TM-score = 0.55), especially along the α helices in the C-terminus; a region previously shown to stimulate histone methyltransferase activity in SUZ12 (Birve et al., 2001) (pos. 580 to 612) is highlighted by a black bar, and individual amino acids important for PRC2 assembly (Birve et al., 2001) are shown by black arrows.

The functional core of PRC2 is highly conserved in animals and consists of four subunits: the H3K27 methyltransferase EZH2/1 and associated proteins EED, SUZ12, and RBBP4/7 (Bieluszewski et al., 2021; Glancy et al., 2021; Simon and Kingston, 2009) (Figures 1A and 1B). SUZ12 interacts with all members of the PRC2 core to form two distinct lobes (Chen et al., 2018; Glancy et al., 2021; Kasinath et al., 2018). The N-terminal region of SUZ12 together with RBBP4/7 forms the targeting lobe, which contributes to the recruitment and regulation of PRC2, and serves as a platform for cofactor binding (Chen et al., 2018; Kasinath et al., 2018). The region of SUZ12 included in this lobe contains five motifs and domains: zinc-finger binding (ZnB), WD-domain binding 1 (WDB1), C2 domain, zinc finger (Zn), and WD-domain binding 2 (WDB2) (Chen et al., 2018; Kasinath et al., 2018) (Figure 1B). The C-terminal region of SUZ12 contains a VEFS domain (Figure 1B), which associates with EZH2/1 and EED to form the catalytic lobe of PRC2 (Chen et al., 2018; Kasinath et al., 2018). Thus, SUZ12 is critical for the assembly, integrity, and function of PRC2, in agreement with the conservation of SUZ12 as a core PRC2 component in animals (Figure 1A).

Genetic and biochemical studies in the nematode *C. elegans* revealed a functional PRC2 complex without an apparent SUZ12 ortholog (Ahringer and Gasser, 2018; Bender et al., 2004; Capowski et al., 1991; Gaydos et al., 2014; Ketel et al., 2005; Korf et al., 1998; Xu et al., 2001). The components of this complex were originally defined by specific maternal-effect sterile (*mes*) mutations that cause defects in germline development and silencing of the X chromosome in the hermaphrodite germline (Capowski et al., 1991; Garvin et al., 1998). Molecular characterizations revealed that MES-2 and MES-6 are homologs of the Polycomb group proteins EZH2/1 and EED, respectively (Xu et al., 2001). MES-2 (EZH2/1) and MES-6 (EED) form a protein complex with MES-3, and all three components are required for histone H3K27 methyltransferase activity *in vivo* and *in vitro* (Ahringer and Gasser, 2018; Bender et al., 2004; Gaydos et al., 2014; Korf et al., 1998; Xu et al., 2001). Despite the functional similarity with the PRC2 core, MES-3 appeared to lack obvious motifs or sequence similarity to SUZ12 or RBBP4/7 and therefore has been considered a *C. elegans* specific subunit (Ahringer and Gasser, 2018; Bender et al., 2004; Ketel et al., 2005; Paulsen et al., 1995; Xu et al., 2001). Consequently, PRC2 in *C. elegans* and in animals are considered functional analogues, despite a seemingly divergent subunit composition (Ahringer and Gasser, 2018; Bender et al., 2004; Ketel et al., 2005; Xu et al., 2001). In-depth sequence comparisons have recently turned up surprising homologies (Yoshida et al., 2019), which prompted us to investigate whether MES-3 could be a highly diverged homolog of SUZ12 instead of a *C. elegans* specific invention.

## Results

### MES-3 is a highly divergent ortholog of the canonical PRC2 component SUZ12

To identify MES-3 homologs in animals, we used unbiased sensitive profile-vs-profile searches to query the predicted human proteome with MES-3 and query the worm proteome with SUZ12. Surprisingly, we recovered a consistent but insignificant bidirectional match between SUZ12 and MES-3 (16% identity; Figure 1C) that is located at approximately the same regions in both proteins and covers 223 amino acids in MES-3. This region in SUZ12 spans part of the ZnB motif, the complete WDB1 motif, and most of the C2 domain (Figures 1B and 1C). Notably, the conserved RBBP4/7 binding site of SUZ12 (Schmitges et al., 2011) is also present in MES-3 (MES-3, pos. 108-113; FLxRx[VL]) as well as a conserved glycine (MES-3, pos. 299) (Figure 1C); a missense mutation of this glycine in *Drosophila* leads to a partial loss-of-function phenotype (Birve et al., 2001; Rai et al., 2013). Therefore, we conclude that the N-terminal regions of SUZ12 and MES-3 share extended sequence similarity including residues previously shown to be critical for function, suggesting that these two proteins are homologs. However, the profile-to-profile searches did not detect similarity between the C-terminal sequence of MES-3 and the SUZ12 domain that mediates EZH2 and EED interaction (Chen et al., 2018; Kasinath et al., 2018) (Figure 1B).

Protein structure is typically more conserved than primary sequence and better allows detection of diverged homologs (Sanchez-Pulido and Ponting, 2021). Because the protein structure of MES-3 is not yet experimentally resolved, we used deep-learning driven protein structure prediction of both MES-3 and SUZ12 with Alphafold2 (Jumper et al., 2021). The SUZ12 structure has six functional motifs and domains that were predicted with high precision as they resemble the experimentally determined structure (RMSD = 0.56–1.14; global TM-score = 0.70; global Dali *Z* score = 14.8 Figures S1A–S1E). Like SUZ12, the predicted MES-3 structure is partially disordered (Figures 1D; Figures S1F–S1H), but nevertheless has a globular N-terminal region mainly formed by β-sheets and a C-terminal region mainly formed by α-helices (Figures 1D and 1E), and both regions were modeled with high confidence (Figure S1G). Interestingly, the C2 domain of SUZ12 shares significant structural similarity with the N-terminal structural regions of MES-3 (Figures 1D and 1E; Figure S1I; RMSD = 1.607; TM-score = 0.60; Dali *Z* score = 11.6), corroborating the profile-vs-profile results (Figure 1C). The structural similarity (MES-3, pos. 150-365) extends beyond the region of shared sequence similarity identified earlier (MES-3, pos. 150-312) and thus encompasses the complete C2 domain (Figure 1D; Figure S1I). Nevertheless, we also observed some differences in the predicted structures such as the occurrence of an unmatched α helix in MES-3 (Figure 1E; Figure S1I) or the absence of amino acids in MES-3 known to be involved in the interaction between SUZ12 and RBBP4/7 (e.g., SUZ12, pos. R196 (Chen et al., 2018)).

Likewise, we observed structural similarity between the C-terminal domain of MES-3 and the VEFS domain in SUZ12 (Figures 1B, 1D, 1F, and 1G; RMSD = 3.676; TM-score = 0.55; Dali *Z* score = 8.3). The MES-3 VEFS-like region is considerably shorter compared with SUZ12 and lacks amino acids that are thought to be involved in the stimulation of histone methyltransferase activity (SUZ12, pos. 580 to 612 (Rai et al., 2013)), specifically SUZ12 E610 and K611 (Rai et al., 2013), which are invariant in plants, animals, and fungi (Figure 1G; Figure S1J). By contrast, several bulky or hydrophobic aromatic residues whose deletion impacts PRC2 assembly (Birve et al., 2001; Rai et al., 2013) are conserved, e.g., SUZ12, pos. F639, I647, L652, and F656 can be aligned to identical residues in superposition of the SUZ12 and MES-3 VEFS predicted structures (Figure 1G; Figure S1J). This suggests that even though the overall sequence similarity is very low, the VEFS domain is overall well conserved in MES-3.

### Similar to SUZ12, MES-3 provides a structural scaffold for PRC2

**Figure 2:**
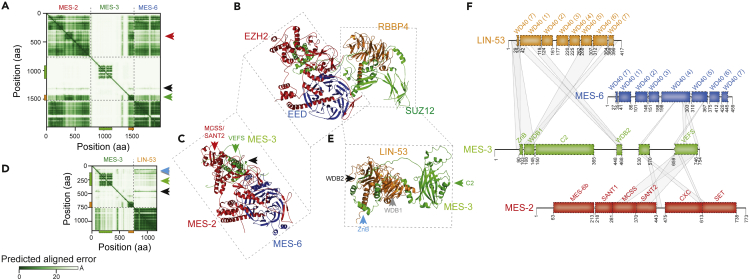
MES-3 provides a structural scaffold for PRC2 in *C. elegans* (A). The predicted aligned error (in Å) of MES-3 co-folded with MES-2 and MES-6 is shown as a heatmap and is consistent with association of MES-3 with MES-2 and MES-6 in the C-terminal regions of MES-3, which overlaps with the predicted VEFS domain. (B). Experimentally resolved human core PRC2 (rcsbpdb:6WKR (Kasinath et al., 2021)) highlights interactions between SUZ12 and RBBP4 as well as SUZ12 and EZH2 and EED. (C). Predicted *C. elegans* core PRC2 is formed by MES-2, MES-3, and MES-6. The corresponding region in human PRC2 is highlighted, as well as the position of the MES-3 VEFS domain (green triangle, see a.) and the MES-2 MCSS/SANT2-like region (red triangle, see a.), as well as a short central region of MES-3 (pos. 530-570) that associates with multiple regions in MES-2 and MES-6 (black triangle, see a.). For clarity, only regions of MES-3 interacting with MES-2 and MES-6 are shown (MES-3, pos. 1-530 and 570-640 are hidden). (D). The predicted aligned error (in Å) of MES-3 co-folded with LIN-53 is shown as a heatmap and reveals association between the N-terminal region of MES-3 and LIN-53. (E). Predicted MES-3 with LIN-53 complex. The corresponding region in human PRC2 is highlighted, as well as the MES-3 C2 domain (green triangle, see d.) and regions surrounding the C2 domain that engage in association with LIN-53 (WDB2, black triangle; WDB1, gray triangle; ZnB, light-blue triangle; see d.). For clarity, only regions of MES-3 interacting with LIN-53 are shown (MES-3, pos. 1-80, 365-415, and 470-754 are hidden). (F). Schematic representation of MES-3 and its predicted interactions with MES-2, MES-6, and LIN-53. The positions of protein domains/motifs in MES-2, MES-6, and LIN-53 were inferred via structural alignments of the predicted structures to those of the experimentally determined human EZH2, EED, and RBBP4 (Chammas et al., 2020; Kasinath et al., 2021). MES-3 domains/motifs are indicated as in Figure 1 with the addition of the central MES-2/MES-6 interacting region; domains/motifs shown are WD-domains (WD40) in MES-6 and LIN-53, and MES-6 binding (MES-6b), Swi3, Ada2, N-CoR and TFIIIB DNA-binding domain 1 like (SANT1), Motif connecting SANT1 and SANT2 (MCSS), SANT2, CXC, and the Su(var)3-9 , EZ and Trx domain (SET) in MES-2 (Chammas et al., 2020). We note that the region around the potential MCSS/SANT2 domains in MES-2 is substantially diverged compared with EZH2, yet still displays considerable structural similarity.

MES-3 together with MES-2 (EZH2) and MES-6 (EED) forms a stable heterotrimeric protein complex (Ketel et al., 2005; Xu et al., 2001). To identify potential interaction surfaces of MES-3, we used Alphafold2 (Evans et al., 2022; Jumper et al., 2021) to generate high-quality structure predictions for MES-2 and MES-6 monomers (Figures S2A–S2l) as well as the trimeric MES-2, MES-3, and MES-6 core complex (Figures 2A and 2C; Figure S2M). As in human PRC2 (Chammas et al., 2020; Chen et al., 2018; Kasinath et al., 2018, 2021) (Figure 2B), the C-terminal VEFS domain of MES-3 is predicted to be associated with MES-2 and MES-6 (Figures 2A, 2C, and 2F). Even though the VEFS domain in MES-3 is shorter than in SUZ12 (Chammas et al., 2020) (Figure 1G), it interacts with a region of MES-2 (MES-2, pos. 300 to 450; Figures 2C and 2F) that in EZH2 comprises the MCSS and the SANT2 domain, which together with VEFS stimulate histone methyltransferase activity (Chammas et al., 2020; Rai et al., 2013). Although these elements were previously noted to be absent in MES-2 (Ketel et al., 2005), our comparison suggests that this region in MES-2 shows potentially similar structural elements yet considerable sequence divergence compared with EZH2. We also identified a short region of MES-3 (MES-3, pos. 530-570) that is associated with regions in both MES-2 and MES-6 (Figures 2C and 2F). The N-terminal region of SUZ12 together with RBBP4/7 forms the targeting lobe (Chammas et al., 2020; Chen et al., 2018; Kasinath et al., 2021), and thus we sought to predict interaction surfaces between MES-3 and LIN-53, one of two closely related seven WD40-repeat proteins, and the protein that most likely retained the ancestral RBBP4/7 function (Figure 2D; Figure S2N). Similar to human PRC2 (Chammas et al., 2020; Chen et al., 2018; Kasinath et al., 2021) (Figure 2B), we observed interactions of the WDB1 domain with the WD40 repeats at the N- and C-terminus of LIN-53 (Figures 2E and 2F). We also identified a second short region in MES-3 (MES-3, pos. 448-468) that interacts with N-terminal WD40 repeats in LIN-53, resembling the interaction of WDB2 in human PRC2 (Chen et al., 2018; Kasinath et al., 2021) and thus MES-3 WDB1 and WDB2 likely wrap around WD40 repeats of LIN-53 (Figure 2F), which in human PRC2 inhibits H3K4 binding of RBBP4/7 (Chen et al., 2018).

## Discussion

Here, we provide evidence that MES-3, even though diverged, structurally resembles SUZ12 in two large regions that are involved in mediating EZH2/1, EED, and RBBP4/7 binding. It is therefore conceivable that, similarly to SUZ12 (Chen et al., 2018; Kasinath et al., 2018), MES-3 is critical in assembling and maintaining a functional PRC2. The uncovered sequence and structural similarities as well as the peculiar complementary phylogenetic profiles strongly suggest that MES-3 and SUZ12 are in fact orthologs, albeit that MES-3 has undergone rapid sequence divergence and loss of crucial amino acid motifs as well as the Zn domain. Besides, *C. elegans* specific evolution of the PRC2 assembly and architecture is likely to also play a role. The PRC2 catalytic lobe, which consist of the SUZ12 VEFS domain in association with EZH2 and EED (Chen et al., 2018; Kasinath et al., 2018), appears the most structurally conserved part of *C. elegans* PRC2. The most notable differences between SUZ12 and MES-3 reside in the N-terminal targeting lobe, which mediates interaction with RBBP4/7, nucleosomes, and accessory proteins (Chen et al., 2018; Kasinath et al., 2018). From flies to humans, distinct PRC2.1 and PRC2.2 sub-complexes can be distinguished that differ in associated accessory proteins and have specialized functions (Chammas et al., 2020; Hauri et al., 2016; Kasinath et al., 2021; Margueron and Reinberg, 2011). For example, the accessory proteins JARID2 and AEBP3 form part of PRC2.2 and mediate interaction with H2AK119ub1 (Kasinath et al., 2021), the product of the PRC1 E3 ubiquitin ligase complex (Margueron and Reinberg, 2011). Although homologs of JARID2 and other accessory proteins remain to be identified in *C. elegans*, the reported candidate PRC1 components are not required for germline development, in contrast to PRC2 (Karakuzu et al., 2009). This may explain the lack of conservation of the Zn domain, which in SUZ12 forms part of the JARID2 interaction surface (Chen et al., 2018). Additional characterizations of *C. elegans* PRC2 and its accessory proteins will be needed to further substantiate this hypothesis.

The here described similarities and differences between SUZ12 and MES-3 should facilitate further experiments to elucidate the specific mechanisms by which MES-3 acts in PRC2 in *C. elegans*. Our work joins a rapidly growing set of *in silico* predictions of previously undetected homologies made possible by unprecedented advances in deep-learning driven structure prediction (Bayly-Jones and Whisstock, 2021; Sanchez-Pulido and Ponting, 2021).

### Limitation of the study

We capitalized on recent advancements in computational prediction approaches that enable to derive high-quality structures of protein monomers or multimers (Evans et al., 2022; Jumper et al., 2021), which enables to study protein function and evolution at unprecedented scale (Bayly-Jones and Whisstock, 2021; Sanchez-Pulido and Ponting, 2021). We demonstrate that MES-3 is a diverged ortholog of SUZ12, and that MES-3 may associate with MES-2, MES-6, and LIN-53, similar to the orthologous proteins in human PRC2. However, this study is strictly based on computational predictions, and thus further experiments will be needed to support our predictions and to elucidate how MES-3 functions in *C. elegans* PRC2. This may come, for instance, from resolving the structure of PRC2 in *C. elegans* as well as from genetic engineering experiments of MES-3 in which predicted conserved amino acids and interaction surfaces are modulated, in combination with biochemical and phenotypic characterization.

## STAR★Methods

### Key resources table

**Table:** 

REAGENT or RESOURCE	SOURCE	IDENTIFIER
**Deposited data**		

*C. elegans* MES-3 sequence	Uniprot DB	Q10665
*C. elegans* MES-2 sequence	Uniprot DB	O17514
*C. elegans* MES-6 sequence	Uniprot DB	Q9GYS1
*C. elegans* LIN-53 sequence	Uniprot DB	P90916
Human SUZ12 sequence	NCBI	NP_056170.2
Human SUZ12 sequence	Uniprot DB	Q15022
Human PRC2-AEBP2-JARID2 structure	PDB	6WKR

**Software and algorithms**		

Orthofinder (v2.1.2)	Emms and Kelly, 2015	https://github.com/davidemms/OrthoFinder
Broccoli (v1.0)	Derelle et al., (2020)	https://github.com/rderelle/Broccoli
EggNOG (v4.5.1)	Huerta-Cepas et al., (2016)	http://eggnog5.embl.de/#/app/home
SonicParanoid (v1.3.0)	Cosentino and Iwasaki (2019)	https://gitlab.com/salvo981/sonicparanoid2
HHPRED (June 2^nd^, 2021)	Gabler et al., (2020)	https://toolkit.tuebingen.mpg.de/tools/hhpred
Alphafold2 (v2.1)	Jumper et al., (2021)	https://github.com/deepmind/alphafold
Pymol (v2.5.2)	Schrödinger, LLC.	https://pymol.org/2/
pdb-tools (v2.4.1)	Rodrigues et al., (2018)	https://github.com/haddocking/pdb-tools
TM-align (v20190822)	Zhang and Skolnick, 2005	https://zhanggroup.org/TM-align/
Dali (v5)	Holm and Sander (1993)	http://ekhidna2.biocenter.helsinki.fi/dali/
IUPRed3 (v3)	Erdős et al. (2021)	https://iupred3.elte.hu

### Resource availability

#### Lead contact

Further information and requests for resources and data should be directed to and will be fulfilled by the lead contact, Michael F. Seidl (m.f.seidl@uu.nl).

#### Materials availability

This study did not generate new unique reagents.

### Method details

#### Sequence similarity searches

We predicted the occurrence of orthologous sequences of the PRC2 core components in diverse Metazoans based on previously computed ortholog assignments from Orthofinder (Emms and Kelly, 2015), Broccoli (Derelle et al., 2020), EggNOG (Huerta-Cepas et al., 2016), and SonicParanoid (Cosentino and Iwasaki, 2019) on a set of reference animal genomes (Deutekom et al., 2020). We manually inspected these orthology assignments based on consistency, which was further corroborated as the predicted occurrences of PRC2 subunits inferred from our assignments consistently matched those published previously (e.g. (Schuettengruber et al., 2017; Sharaf et al., 2021)).

For sensitive profile-vs-profile searches, we used HHPRED as provided on the MPI Bioinformatics Toolkit server (Gabler et al., 2020). We performed one search using *C. elegans* MES-3 (uniport: Q10665; MES3_CAEEL) as query and profiles of the human proteome as database, which found as best hit the human SUZ12 protein (ncbi:NP_056170.2) with an e-value 860 and score 38.4. Next, a reciprocal search was performed with human SUZ12 as query and the *C. elegans* proteome as database, which found as best hit MES-3 with an e-value of 970 and score of 28.7; human SUZ12 and *C. elegans* MES-3 are thus in a reciprocal best hit relation of sequence profiles, which is a clear indication for orthology (Szklarczyk et al., 2012).

#### SUZ12 and MES-3 structure prediction and comparison

We predicted the protein structures of SUZ12 (uniprot:Q15022) and MES-3 (uniprot:Q10665) using a local Alphafold2 (Jumper et al., 2021) instance (version 2.1; five monomer models (Jumper et al., 2021) as well as model 2 ptm (Jumper et al., 2021) to obtain the predicted aligned errors, full genetic database, and maximum template date: 01-11-2021). We compared the here predicted with the experimentally determined (rcsbpdb:6WKR-A (Kasinath et al., 2021)) structure of SUZ12 using the sequence-independent structure comparisons with super, which is implemented in pymol. Motifs in SUZ12 were selected based on amino acid coordinates (Chammas et al., 2020) (amino acid coordinates are shown in Figure 1B), and extracted from pdb files using pdb-tools (Rodrigues et al., 2018); extracted motifs and domains were subsequently structurally imposed onto the predicted MES-3 using super and/or cealign on the C-alpha atoms, and the root mean square deviation (RMSD; presented in Å) between the structures was used as a measure of structural divergence; an RMSD below 2 Å is generally considered to indicate two very similar structures. We furthermore used TM-align (version 20190822; default parameters) (Zhang and Skolnick, 2005) as well as Dali (Holm and Sander, 1993) to obtain sequence-independent structure alignments between SUZ12 and MES-3 (sub)structures; TM align TM-scores 0.5 < x < 1 and Dali Z-scores > 2 typically indicate similar folds. Disordered regions in the protein sequences were predicted using IUPRed3 (default settings) (Erdős et al., 2021). The protein (sub)structures were visualized using pymol, and the data visualization was performed with python seaborn.

#### PRC2 complex structure prediction and comparison

We predicted the monomeric structures of the members of the PRC2 core complex, MES-2 (EZH2; uniprot:O17514), MES-6 (EED; uniprot:Q9GYS1), and LIN-53 (RBBP4; uniprot:P90916), with Alphafold2 and compared these monomeric predictions with experimentally predicted structure of human PRC2 members (rcsbpdb:6WKR (Kasinath et al., 2021)) as described above. We predicted multi-chain PRC2 complex interactions of MES-2, MES-3, and MES-6 as well as MES-3 and LIN-53 using Alphafold2-multimer (Evans et al., 2022) (version 2.2; five multimer models (Evans et al., 2022) with each five seeds, full genetic database, and maximum template date: 01-11-2021). Predicted multimer models were compared with monomer models using super as well as TM-align (Zhang and Skolnick, 2005) as described above, and interaction interfaces between protein pairs within complexes were predicted using pymol (default settings).

## Data Availability

• This paper analyzes existing, publicly available data. These accession numbers for the datasets are listed in the key resources table. • This paper does not report original code. • Any additional information required to reanalyze the data reported in this paper is available from the lead contact upon request.
